# Protracted delay in taste sensation recovery after surgical lingual nerve repair: a case report

**DOI:** 10.1186/1752-1947-7-77

**Published:** 2013-03-18

**Authors:** Kenji Seo, Yuji Inada, Makoto Terumitsu, Tatsuo Nakamura, Keiji Shigeno, Yutaka Tanaka, Tatsuru Tsurumaki, Shigenobu Kurata, Hitoshi Matsuzawa

**Affiliations:** 1 Division of Dental Anesthesiology, Department of Tissue Regeneration and Reconstruction, Niigata University Graduate School of Medical and Dental Sciences, Course for Oral Sciences, 2-5274 Gakkocho-dori, Chuo-ku, Niigata, 951-8514, Japan; 2 Department of Orthopaedic Surgery, Inada Hospital, 46 Omori-cho, Nara, 630-8131, Japan; 3 Department of Bioartificial Organs, Institute for Frontier Medical Sciences, Kyoto University, 53 Kawahara-cho, Sakyo-ku, Kyoto, 606-8507, Japan; 4 Division of Dental Anesthesiology, Niigata University Medical and Dental Hospital, University of Niigata, 1-754, Asahimachi-dori, Chuo-ku, Niigata, 951-8520, Japan; 5 Center for Integrated Human Brain Science, University of Niigata, 1-757, Asahimachi-dori, Chuo-ku, Niigata, 951-8585, Japan

**Keywords:** Lingual nerve, Long observation, Peripheral nerve injury, Polyglycolic acid-collagen tube, Taste

## Abstract

**Introduction:**

Lingual nerve injury is sometimes caused by dental treatment. Many kinds of treatment have been reported, but many have exhibited poor recovery. Here the authors report changes in somatosensory and chemosensory impairments during a long-term observation after lingual nerve repair.

**Case presentation:**

A 30-year-old Japanese woman claimed dysesthesia and difficulty eating. Quantitative sensory test results indicated complete loss of sensation in the right side of her tongue. She underwent a repair surgery involving complete resection of her lingual nerve using a polyglycolic acid tube containing collagen 9 months after the injury. A year after the operation, her mechanical touch threshold recovered, but no other sensations recovered. Long-term observation of her somatosensory and chemosensory function after the nerve repair suggested that recovery of taste sensation was greatly delayed compared with that of somatosensory function.

**Conclusion:**

This case shows characteristic changes in somatosensory and chemosensory recoveries during 7 postoperative years and suggests that taste and thermal sensations require a very long time to recover after repair surgery.

## Introduction

We previously reported the 1-year prognosis of lingual nerve repair using a polyglycolic acid tube containing collagen (PGA-collagen tube) (Kyoto University, Kyoto, Japan) in a 30-year-old woman [1]. This patient underwent surgery 9 months after an injury because of dysesthesia and difficulty eating. Quantitative sensory test results indicated complete loss of sensation in the right side of her tongue. A year after the operation, the mechanical touch threshold recovered, but no other sensations recovered. A 7-year observation revealed that taste sensation required a long time to recover. We here present temporal changes in several types of sensations during a period of 7 years after the operation and discuss the characteristic recovery features of different sensations.

## Case presentation

A 30-year-old Japanese woman underwent extraction of an impacted wisdom tooth of the right mandible. Almost 1 week later, she noticed prolonged numbness in the right lingual area. Furthermore, she began to experience difficulty talking and eating for several weeks because of gradually increasing chronic lingual pain. She was diagnosed with complete sensory loss in the right lingual nerve region. The fungiform papillae were completely lost on the right side of her tongue. A quantitative sensory test involving brush stroke perception, mechanical touch threshold, two-point discrimination (2PD), thermal perception, and taste sensation was conducted repeatedly before and after the operation. Thermal sensation was assessed by determining whether the patient could recognize warm or hot after a thermal applicator (TI-3101; KGS Corporation, Saitama, Japan) was touched to her tongue for several seconds; this estimated her ability to perceive a temperature of 42°C or higher. Taste examination was evaluated by electrogustometry (TR-06®; Rion Co. Ltd., Tokyo, Japan) and the filter-paper disk method (Sanwa Kagaku Kenkyusho Co. Ltd). In this method, circular-cut filter papers with a 5mm diameter were soaked in three solutions of different concentrations. They consisted of 0.3%, 2.5%, 10%, 20%, and 80% sucrose; 0.3%, 1.25%, 5%, 10%, and 20% sodium chloride; and 0.02%, 0.2%, 2%, 4%, and 8% tartaric acid. Each was placed onto the anterior two-thirds of her tongue, which is innervated by the chorda tympani nerve, in order of concentration. The patient was asked what tastes among sweet, salty, and sour she could perceive, and the taste threshold was then examined. Psychometric tests, including the Hospital Anxiety and Depression Scale, the State-Trait Anxiety Inventory, and a self-rating questionnaire for depression, were administered prior to the treatment. There were no abnormal findings in any test. Prior to the operation, informed consent was obtained from the patient. Surgery was conducted under general anesthesia 9 months after the injury. The medial and distal ends of the lingual nerve were separated by >40mm intervals. Neuromas were removed from each end of the lingual nerve, and the freshly cut ends were inserted on the inside and fixed to both ends of a PGA-collagen tube (length, 50mm; outer diameter, 5mm) under microsurgery.

**Figure 1:**
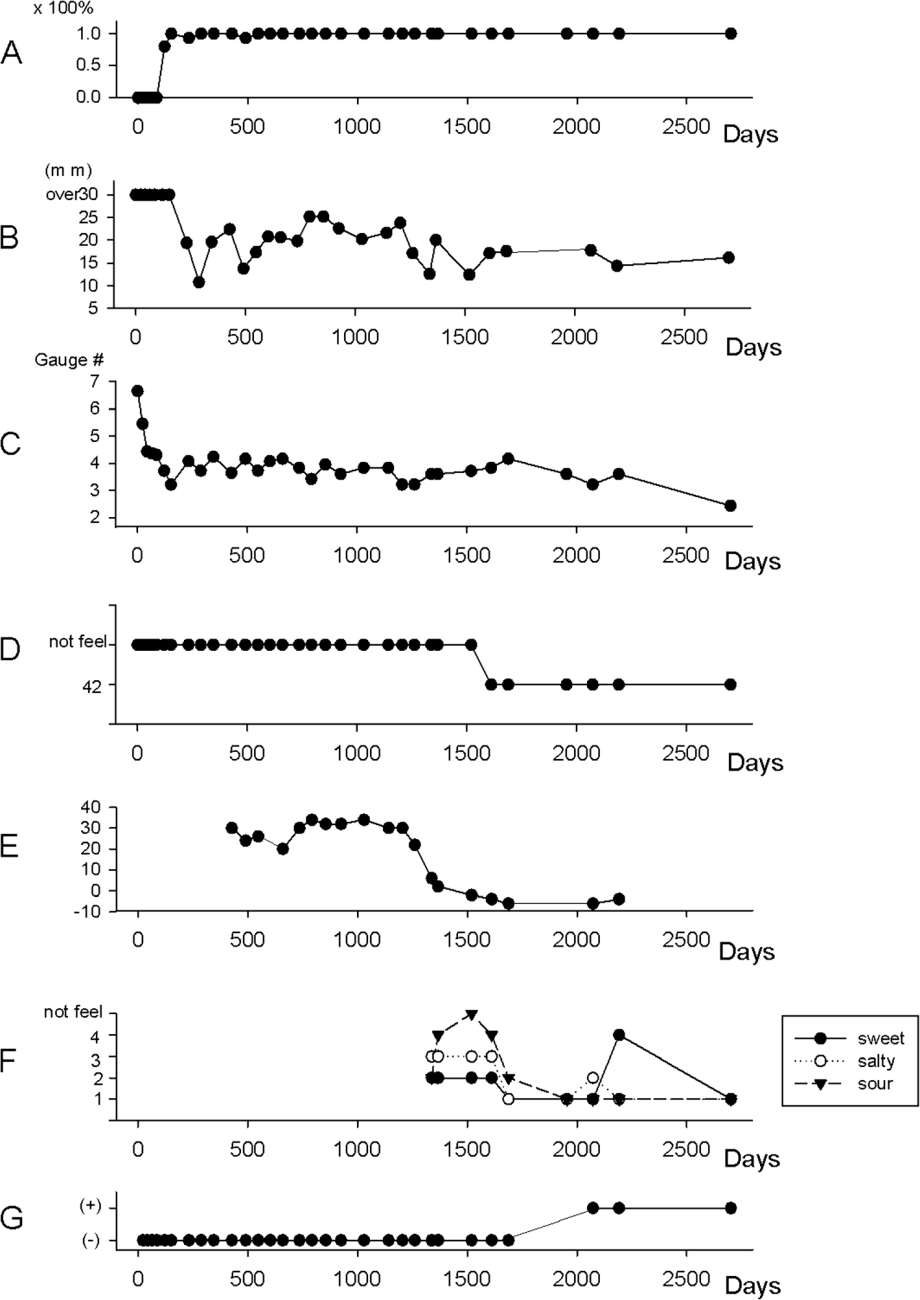
**Temporal changes in the recovery of multiple sensations after the operation. (A)** Brush stroke perception. The brush stroke perception rate improved shortly after the operation. **(B)** Two-point discrimination. Two-point discrimination remained at a high level (>30mm) for almost 1 year postoperatively, but it then decreased and remained at a lower level. **(C)** Mechanical touch threshold. The threshold expressed by the von Frey monofilament gauge number improved to a level lower than the reported normal value within 1 year postoperatively. **(D)** Thermal perception. The thermal threshold remained at more than 50°C for 2 years postoperatively, but then improved to 50°C. Recovery to the normal range took 5 years. **(E)** Electric taste threshold of the tongue on the injured side. Within 1 year postoperatively, it remained high at >34dB. It subsequently began to decrease and reached the normal range 5 years postoperatively. **(F)** Chemical tests of the taste threshold in the tongue. Ordinates of sweet: 1, 2, 3, 4, and 5 indicate 0.3%, 2.5%, 10%, 20%, and 80% sugar solution, respectively. Ordinates of salty: 1, 2, 3, 4, and 5 indicate 0.3%, 1.25%, 5%, 10%, and 20% sodium chloride solution, respectively. Ordinates of sour: 1, 2, 3, 4, and 5 indicate 0.02%, 0.2%, 2%, 4%, and 8% tartaric acid, respectively. The three kinds of taste threshold (sweet, salty, and sour) remained high, and the patient could not detect any tastes. These tastes were detected 4 years postoperatively, and reached an almost normal level around 7 years postoperatively. **(G)** Presence of subjective taste sensation in daily life. Subjective recovery of taste sensation was observed 7 years after surgery.

After the operation, the quality of her daily life activities significantly improved because of the resolution of dysesthesia in the tongue during movement. No occurrence of severe neuropathic pain was observed. Changes in quantitative sensory tests were followed postoperatively (Figure 1). There were no significant changes in any test on the uninjured side during the whole observation period. Recovery in brush stroke sensation began 90 days postoperatively and reached a normal level 150 days later. The 2PD threshold began to improve from approximately 200 days after the operation. The mechanical touch threshold also started to improve early after the operation, similar to the brush stroke perception, but it reached a lower level compared with the reported normal value and did not improve further over 6 years. Recovery of thermal perception of hot sensation took more than 2 years and reached a normal level in almost 5 years. Taste sensation exhibited a different temporal change as determined by a different type of monitoring, and recovery of this sensation showed the least improvement compared with other sensations. For the first 3 years, the patient could not completely detect any taste sensations and electrogustometry did not improve; thus, no chemical testing for taste sensations was performed. However, electrogustometry indicated that improvement began approximately 4 years after the operation, at which point chemical testing using the filter-paper disk method was begun. This method indicated that improvement took more than 4 years, and sour taste showed the slowest recovery. By contrast, recoveries of sweet and salty tastes were relatively earlier than recovery of sour. However, subjective perception of taste differed from the results of these taste examinations. Despite apparent improvements according to other taste examination results, recovery was delayed for more than 1 year following the achievement of normal levels of these measurements. As a result, all taste perceptions required almost 7 years to recover.

**Figure 2:**
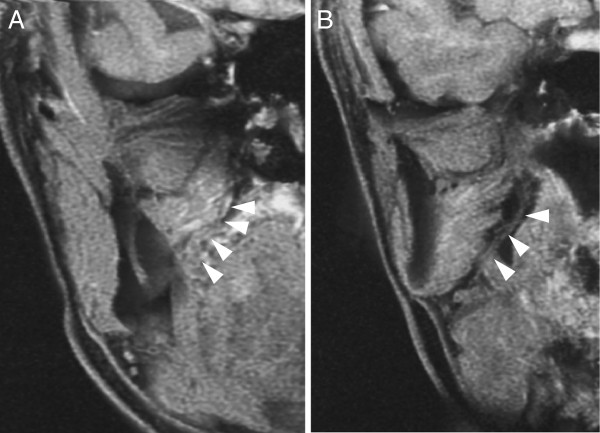
**Coronal image of the regenerated lingual nerve as presented by three-dimensional volume rendering-magnetic resonance neurography taken 7 years postoperatively.** The images were from the anterior **(A)** to the posterior **(B)** sides. White arrows indicate the regenerated lingual nerve.

**Figure 3:**
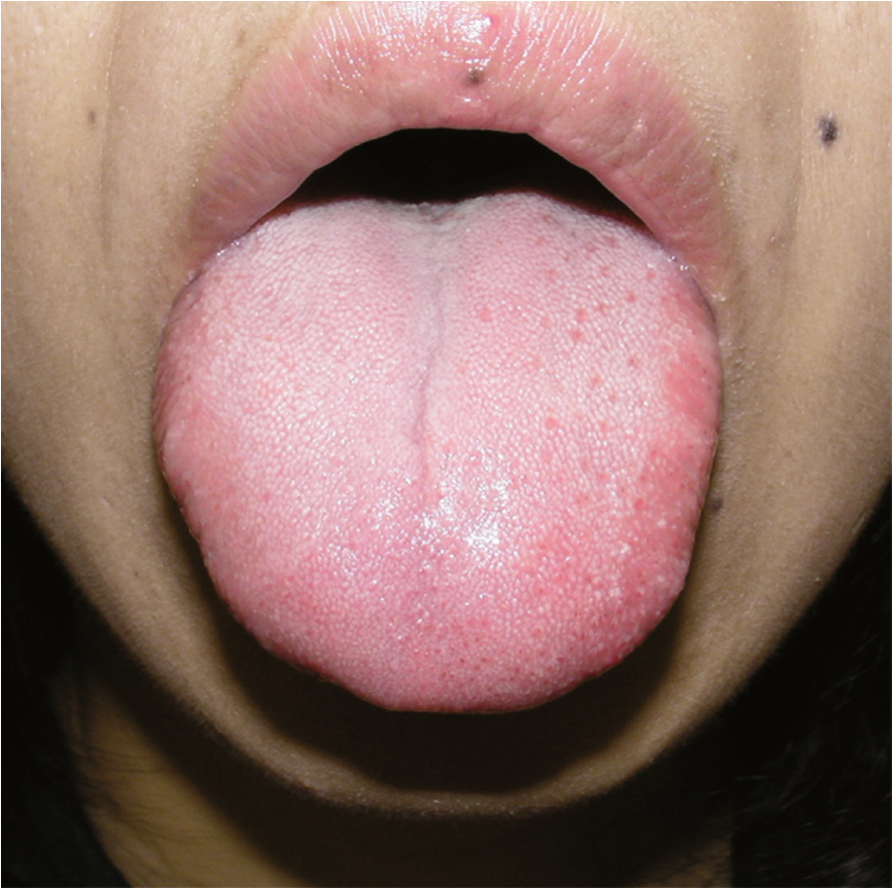
**Representative photograph of the tongue 7 years postoperatively.** The taste buds can be observed on the surface of the regenerated (right) side of the tongue, but their sizes and quantity were smaller than those of the healthy (left) side.

High-resolution three-dimensional volume rendering-magnetic resonance neurography (3DVR-MRN) was conducted 7 years postoperatively when all tongue sensations had recovered [2]. The regenerated lingual nerve could be detected on the coronal images (Figure 2). Clinical observation indicated that the taste buds on the regenerated side of the tongue were smaller in size and quantity than those on the healthy side (Figure 3).

## Discussion

This case indicates that somatosensory function recovered within 1 year after repair, but chemosensory recovery took a much longer period of time. Compared with several previous reports [3,4], our observation period was the longest and showed full chronological changes in both somatosensory and chemosensory recoveries. Furthermore, we demonstrated recovery of the nerve deficit by neurography imaging. This is the first report to certify a nerve gap connection by 3DVR-MRN.

The lingual nerve conveys taste fibers from the chorda tympani, which innervates taste buds of the tongue, and iatrogenic nerve injury may cause loss of gustatory sensation in some cases. A Sunderland class 4 or 5 lingual nerve injury decreases the number of fungiform papillae in the anterior tongue soon after an injury, but surgical nerve repair can increase the number of papillae in 1 year. Although the number of regenerated taste receptors increases within 6 months, functional recovery is reportedly very difficult to achieve [4]. Our macro-observation of the tongue surface revealed that the number of fungiform papillae on the injured side of the tongue decreased compared with that before the repair; by contrast, taste function can be obtained again 6 years postoperatively. This is a different feature in that taste sensitivity is correlated with the number of taste buds [5]. This also means that the number of fungiform papillae can be helpful for diagnosis of complete resection of the lingual nerve, but not for monitoring of the recovery of taste function.

Neurosensory parameters as measured by light touch, brush stroke perception, 2PD threshold, pin-prick sensation, and temperature not involving taste sensation indicate that 86% of patients undergoing nerve repair could achieve functional recovery within 1 year after surgery [3,6]. By contrast, some reports concerning postoperative changes in taste sensation indicated poor recovery, but this was judged in short periods of 1 to 3 years [4,6,7]. They reported that whole-mouth taste can be obtained at a low rate of less than 50% within 1 year after repair [7]. In the present case, changes in taste sensation exhibited objective recovery changes as measured by electrogustometry and the filter-paper disk method. Delayed recovery and a long period of time were required to reach the normal level compared with somatosensory sensations. Moreover, subjective recovery of taste sensation emerged later than objective recovery. This may be related to a central reorganization mechanism involving neural circuitry of the peripheral sensory system. An extraordinarily long observation period is required to assess these changes in the many modalities of taste-related tongue sensation.

In this case, a very long period was required to reach full recovery of chemosensory taste sensation in contrast to the changes in somatosensory function. The cause of this may be related to several factors. The first is the timing of nerve repair. Three months after injury is a critical period within which to expect a better prognosis for functional recovery. Therefore, early repair within 3 months after injury may be an important factor contributing to increased improvement of sensation [6]. However, caution is needed because these reports did not treat for recovery of taste sensation. In this case, a patient underwent surgical repair 9 months after the injury. Her tactile sensation recovered as previously reported [1]. This is consistent with other reports. The second factor is the length of the nerve deficit. A nerve gap deficit should be less than 3cm for increased improvement of sensory recovery [4]. Although we used a PGA-collagen tube to connect nerve gaps, good regeneration of nerve gaps occurred. This method is characteristic of little possibility for neuroma recurrence, which differs from the direct suture method [8]. The prognosis may depend on the surgical procedure; for example, autogenous nerve graft or tubulization. The nerve deficit distance was favorable at less than 3cm for the direct suture method [9], but this tube has the potential to regenerate even in cases of deficits longer than 3cm but shorter than 8cm [10]. Therefore, the length of the nerve deficit may be an important factor affecting the recovery period.

## Conclusion

Long-term observation of somatosensory and chemosensory function after nerve repair suggested that recovery of taste sensation was greatly delayed compared with that of somatosensory function.

## Consent

Written informed consent was obtained from the patient for publication of this case report and any accompanying images. A copy of the written consent is available for review by the Editor-in-Chief of this journal.

## Competing interests

The authors declare that they have no competing interests.

## Authors’ contributions

K Seo was a major contributor in writing the manuscript and giving the diagnosis. MT and HM analyzed the magnetic resonance image. YI and TN performed the operation. YT, TT and SK performed the measurements of sensory tests and helped to analyze them. K Shigeno interpreted the patient data. All authors read and approved the final manuscript.
